# Diagnostic reference levels for chest computed tomography in children as a function of patient size

**DOI:** 10.1007/s00247-022-05340-8

**Published:** 2022-04-05

**Authors:** Denise Bos, Sebastian Zensen, Marcel K. Opitz, Johannes Haubold, Kai Nassenstein, Sonja Kinner, Bernd Schweiger, Michael Forsting, Axel Wetter, Nika Guberina

**Affiliations:** 1 Institute of Diagnostic and Interventional Radiology and Neuroradiology, University Hospital Essen, University of Duisburg-Essen, Hufelandstraße 55, 45147 Essen, Germany; 2 Department of Diagnostic and Interventional Radiology, Neuroradiology, Asklepios Klinikum Harburg, Eißendorfer Pferdeweg 52, 21075 Hamburg, Germany

**Keywords:** Chest, Children, Computed tomography, Dosimetry, Radiation safety

## Abstract

**Background:**

Radiation exposures from computed tomography (CT) in children are inadequately studied. Diagnostic reference levels (DRLs) can help optimise radiation doses.

**Objective:**

To determine local DRLs for paediatric chest CT performed mainly on modern dual-source, multi-slice CT scanners as a function of patient size.

**Materials and methods:**

Five hundred thirty-eight chest CT scans in 345 children under 15 years (y) of age (median age: 8 y, interquartile range [IQR]: 4–13 y) performed on four different CT scanners (38% on third-generation and 43% on second-generation dual-source CT) between November 2013 and December 2020 were retrospectively analysed. Examinations were grouped by water-equivalent diameter as a measure of patient size. DRLs for volume CT dose index (CTDI_vol_) and dose-length product (DLP) were determined for six different patient sizes and compared to national and European DRLs.

**Results:**

The DRLs for CTDI_vol_ and DLP are determined for each patient size group as a function of water-equivalent diameter as follows: (I) < 13 cm (*n* = 22; median: age 7 months): 0.4 mGy, 7 mGy·cm; (II) 13 cm to less than 17 cm (*n* = 151; median: age 3 y): 1.2 mGy, 25 mGy·cm; (III) 17 cm to less than 21 cm (*n* = 211; median: age 8 y): 1.7 mGy, 44 mGy·cm; (IV) 21 cm to less than 25 cm (*n* = 97; median: age 14 y): 3.0 mGy, 88 mGy·cm; (V) 25 cm to less than 29 cm (*n* = 42; median: age 14 y): 4.5 mGy, 135 mGy·cm; (VI) ≥ 29 cm (*n* = 15; median: age 14 y): 8.0 mGy, 241 mGy·cm. Compared with corresponding age and weight groups, our size-based DRLs for DLP are 54% to 71% lower than national and 23% to 85% lower than European DRLs.

**Conclusion:**

We developed DRLs for paediatric chest CT as a function of patient size with substantially lower values than national and European DRLs. Precise knowledge of size-based DRLs may assist other institutions in further dose optimisation in children.

## Introduction

The European Commission has emphasised the importance of setting diagnostic reference levels (DRLs) for paediatric radiographic examinations in its Radiation Protection N° 185 [1]. A comprehensive European and worldwide review of DRLs for paediatric examinations shows that only a few countries have set DRLs for paediatric examinations and that for many examinations there are no national DRLs [1]. Examinations with high radiation exposure, such as computed tomography (CT), are particularly important in radiation protection. DRLs have become an indispensable part of radiation protection; they were first recommended by the International Commission on Radiological Protection (ICRP) in 1991 and incorporated into European legislation in 1997 by the Medical Exposure Directive 97/43/EURATOM [2, 3]. Since the European Council Directive 2013/59/EURATOM, the establishment, regular review and use of DRLs, which are set at the 75^th^ percentile of a dose survey, have been mandatory for member states to optimise radiation protection [4]. The United Kingdom National Radiation Protection Board (NRPB) introduced the concept of achievable dose in 1999 to improve further dose optimisation [5], the National Council on Radiation Protection and Measurements (NCRP) in the United States followed up on this and determined that achievable doses would be set at the median of the dose distribution [6].

It is important to protect children from unnecessary radiation exposure as they are more sensitive to the cancer risk of ionising radiation than adults and have more time to develop cancer after radiation exposure [7–10]. This emphasises the need for analysis of large dose data in children and to advance dose optimisation efforts in children. A major problem is the large variation in patient size in infants and children in the same age group. This partially explains the large variation in radiation dose, in addition to other individual, institutional and national factors, all contributing to large variations across patients, institutions and countries [11–17]. Further, there is little consistency in grouping of patients into age and weight groups [1, 16–18]. This is particularly complicated when patient characteristics such as height and weight are not consistently documented at examination [19]. Following the results of Kanal et al. [20], who determined DRLs for different CT examinations in adults as a function of patient size, we aim to establish local DRLs and achievable doses for paediatric chest CT. To the best of our knowledge, this is the first report of local DRLs and achievable doses as a function of patient size, based on water-equivalent diameter rather than classifications by age and weight [20]. We also aim to compare our local DRLs and achievable doses to national and European DRLs for corresponding age and weight groups.

## Materials and methods

### Patient cohort

In this retrospective study at a high-volume, multi-site radiology centre, we included 538 chest CT scans of 345 children 15 years or younger performed between Nov. 1, 2013, and Dec. 1, 2020. Exams were identified using the Digital Imaging and Communications in Medicine (DICOM) header-based dose monitoring software Radimetrics Enterprise Platform (Bayer AG, Leverkusen, Germany), which collects examinations directly from the internal picture archiving and communication system [21]. Medical information, such as clinical indication and diagnosis, was derived from the radiologic information system. Patient diameter as the average of water-equivalent diameter from each CT acquisition over the entire imaging range (calculated by the dose monitoring software) was used to determine patient size. Only examinations with complete DICOM information, particularly water-equivalent diameter, were considered. Ethical approval was given by the Ethics Committee of the Medical Faculty of the University of Duisburg-Essen (20-9776-BO) and the requirement to obtain informed consent was waived.

### Water-equivalent diameter

The water-equivalent diameter was derived from and automatically calculated by the dose monitoring software. It was defined as the diameter of a circle with an area equal to the image water equivalent area, i.e. the area of foreground pixels weighted according to their radiodensity compared to water. It is usually derived from the localizer, but may alternatively be derived from the axial images [22].

### Computed tomography protocols and scanner

Depending on the clinical question, single-phase chest CT was performed with or without contrast medium. Non-contrast CT examinations could be also performed at low dose, while both non-contrast and contrast chest CT could be performed using a high-pitch technique (flash spiral scan mode). CT of the pulmonary arteries was performed with (*n* = 5) and high-resolution chest CT without contrast (*n* = 1). Clinical indications for paediatric chest CT are shown in Table 2. Scans were performed on four different CT scanners: single-source 128-slice SOMATOM Definition AS + , single-source 128-slice SOMATOM Definition Edge, dual-source 128-slice SOMATOM Definition Flash, and dual-source 192-slice SOMATOM Force (all Siemens Healthineers, Erlangen, Germany). Examinations on dual-source CT scanners were performed in single-source mode. Automatic tube current modulation (CARE Dose 4D; Siemens Healthineers, Erlangen, Germany) was utilised for all CT scans, automatically adjusting tube current to the shape and size of the patient, thus achieving an optimal tube current time product for a preselected image quality. Automatic tube voltage modulation (CARE kV; Siemens Healthineers) provides automatic tube voltage adjustment, including the potential for scans at 70 kV. Additionally, X-CARE (Siemens Healthineers) reduces direct X-ray exposure for the most radiation-sensitive anatomical regions and organs, automatically lowering the tube current for a certain range of projections. SAFIRE (sinogram affirmed iterative reconstruction) and ADMIRE (advanced modeled iterative reconstruction), both Siemens Healthineers, were used for iterative reconstruction algorithms, both with a middle strength (strength 3).

### Dose assessment

Radiation doses were assessed with the aid of a dose monitoring software program [21]. Dose assessments referred to the 32-cm polymethyl-methacrylate body phantom. Radiation doses were reported for the volume CT dose index (CTDI_vol_), dose-length product (DLP), size-specific dose estimates (SSDE) and effective dose. CTDI_vol_ reflects the average radiation exposure per section and DLP reflects the total radiation output for the examination. SSDE are doses at the centre of the scanned region of an individual patient, factoring in the patient’s size [23, 24]. Effective dose is the tissue-weighted sum of the equivalent doses in all tissues and organs and can be used to estimate the stochastic health risk for cancer induction [25]. However, effective dose was intended for use as a protection quantity and was defined neither for patients nor for children as it was intended to be a measure for the radiation risk in adults in occupational health care [25]. Therefore, effective dose is not suitable for assessing the radiation risk in children and should be used for comparison only. Effective dose estimates derived from the dose monitoring software are based on calculations of patient-specific organ doses using Monte Carlo simulations. DRLs were set at the 75^th^ percentile of the dose distribution and achievable doses at the median. Radiation dose assessment did not include topograms or intravenous contrast monitoring.

### Statistics

Kolmogorov–Smirnov and Shapiro–Wilk tests were applied to determine normal distribution of variables. Technical parameters and dose metrics of the chest CT scans within the entire patient cohort were not normally distributed. Within patient size groups, variables were also not consistently normally distributed, so that the nonparametric Kruskal–Wallis test and the post hoc test with Bonferroni correction were applied for further analysis between patient size groups. A Spearman's correlation was run to assess the relationship between water-equivalent diameter and both CTDI_vol_ and DLP. The Mann–Whitney *U* test was performed to test for differences within patient size groups between contrast and non-contrast examinations, and between low-dose and standard examinations, and between examinations with high-pitch technology and examination with standard-pitch. A *P*-value lower than 0.05 was considered statistically significant. Statistical analysis was performed with IBM SPSS Statistics v. 27.0. (IBM Corp., Armonk, NY).

## Results

A total of 538 chest CTs in children 15 years of age and younger were analysed. The median age at the time of the performed examination was 8 years (interquartile range [IQR]: 4–13 years) and 42% of the examinations were performed in female patients (Table 1). The majority (64%) of examinations were performed in oncology patients, since our institute belongs to a large cancer center. Table 2 summarises the different types of cancer amongst the patient cohort. Acute lymphoblastic leukaemia (56 of 342 examinations) and rhabdomyosarcoma (49 of 342 examinations) were the most common cancers for which CT of the chest was performed. Twenty-seven percent of all patients (93 of 345) were examined several times during the study period (Table 1). These examinations varied in terms of protocol type, examination date and patient size. Patients with cancer were the most likely to receive multiple chest CTs (to evaluate pulmonary metastases or to detect lung infiltrates); 12 patients had more than four CT chest scans (maximum: 10 times [*n* = 1]) during the 7-year study period.

**Table 1:** Sample sizes and percentages of patients and examinations by gender, computed tomography (CT) scanner, protocol and paediatric chest CT technique

Characteristic	*n*	Percentage
Patients	345	
Gender (female/male)	155/190	45%/55%
Number of patients with		
One exam	252	73%
Two exams	49	14%
Three exams	20	6%
Four exams	12	3%
More than four exams	12	3%
Total number of CT examinations	538	
on female patients	227	42%
on male patients	311	58%
CT scanner^a^		
SOMATOM Definition Flash	232	43%
SOMATOM Force	205	38%
SOMATOM Definition AS +	96	18%
SOMATOM Definition Edge	5	1%
Chest CT protocol		
without contrast	288	54%
with contrast	250	46%
CT technique		
High-pitch (flash spiral scan mode)	169	31%
Low-dose	164	30%

^a^all: Siemens Healthineers, Erlangen, Germany

**Table 2:** Indications for paediatric chest computed tomography (CT) and types of cancer in oncological patients

	*n*	Percentage
CT indications		
Total	538	
Staging	272	51%
Pulmonary infiltrates	164	30%
Pulmonary structural changes/ interstitial lung disease	57	11%
Trauma	8	1%
Vascular questions including pulmonary embolism	7	1%
Abscess	6	1%
Other questions	24	4%
Examinations in oncological patients	342	64%
Types of cancer		
Acute lymphoblastic leukaemia	56	16%
Rhabdomyosarcoma	49	14%
Lymphoma	45	13%
Ewing sarcoma	35	10%
Other sarcomas	32	9%
Acute myeloid leukaemia	30	9%
Osteosarcoma	20	6%
Nephroblastoma	20	6%
Hepatoblastoma	13	4%
Rhabdoid tumour	11	3%
Other cancers	31	9%

The examinations were mainly performed on modern, second- and third-generation dual-source CT scanners (43% on SOMATOM Definition Flash and 38% on SOMATOM Force) (Table 1). In terms of protocol types, 54% (*n* = 288) were without contrast medium. Thirty-one percent (*n* = 169) of all CT examinations were performed using a high-pitch protocol (flash mode) and 30% (*n* = 164) at low dose (Table 1), with 60% (*n* = 99) of these low-dose examinations performed using a high-pitch technique.

**Fig. 1:**
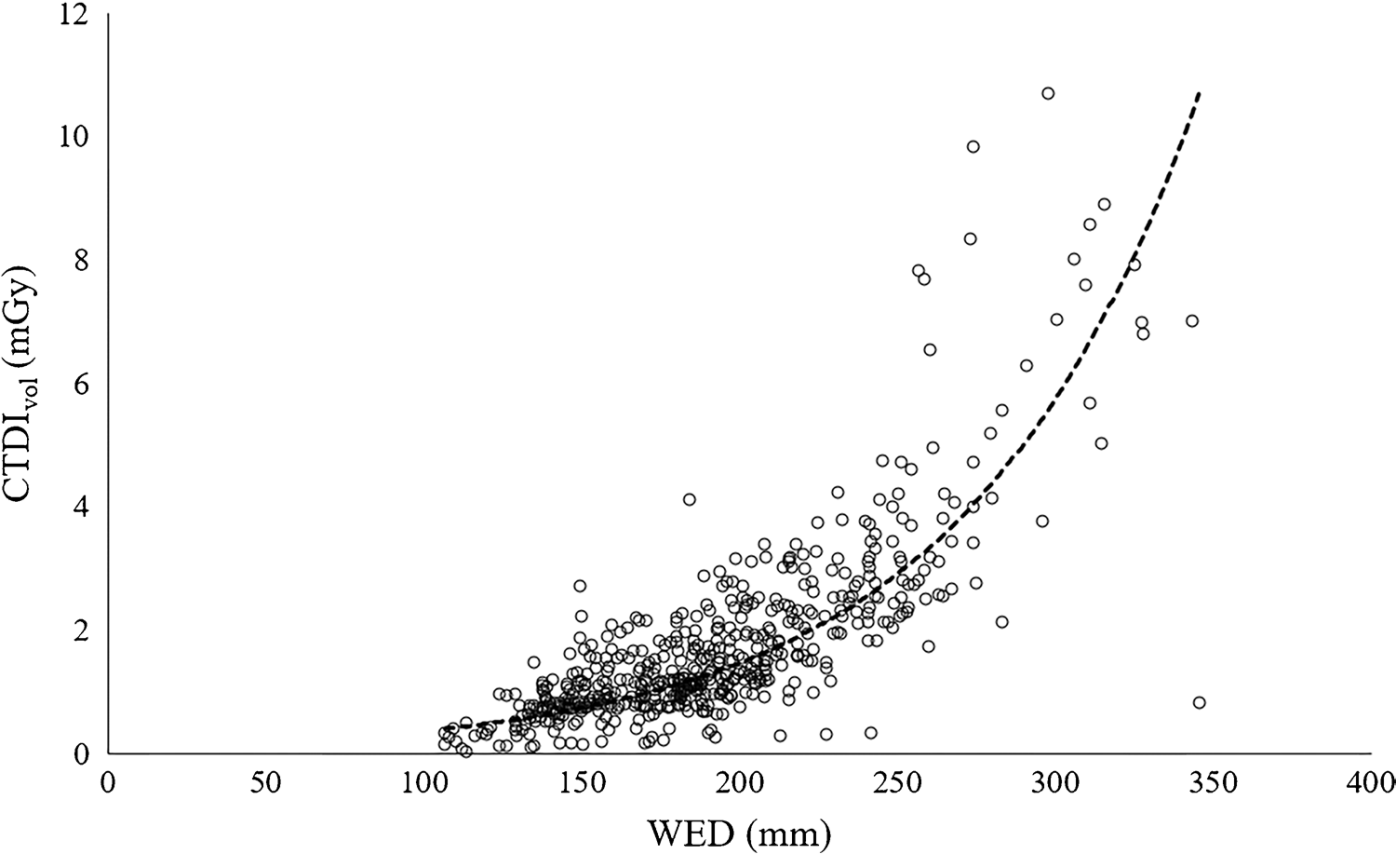
A scatterplot of water-equivalent diameter (WED) against volume computed tomography dose index (CTDI_vol_)

**Fig. 2:**
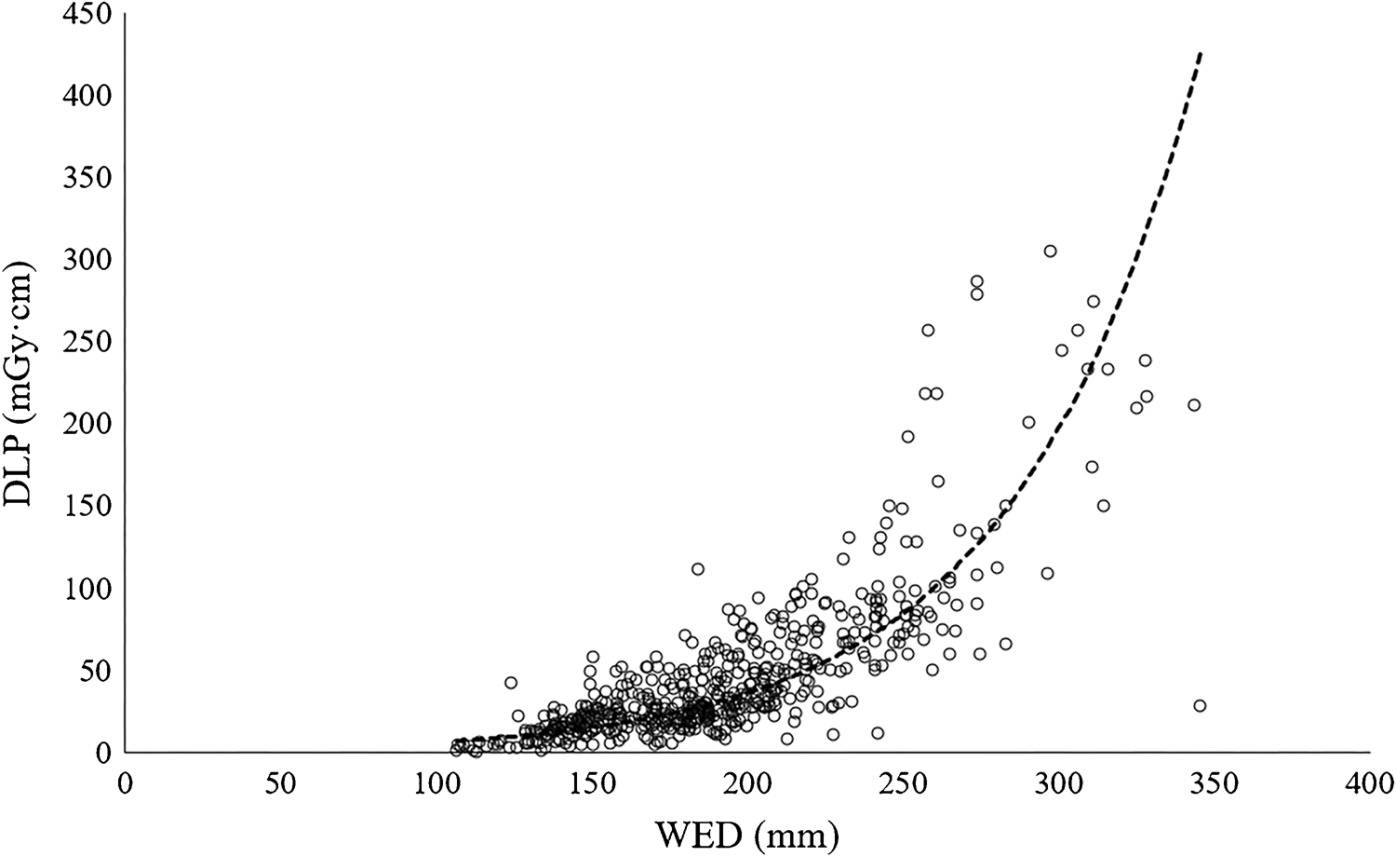
A scatterplot of water-equivalent diameter (WED) against dose-length product (DLP)

**Fig. 3:**
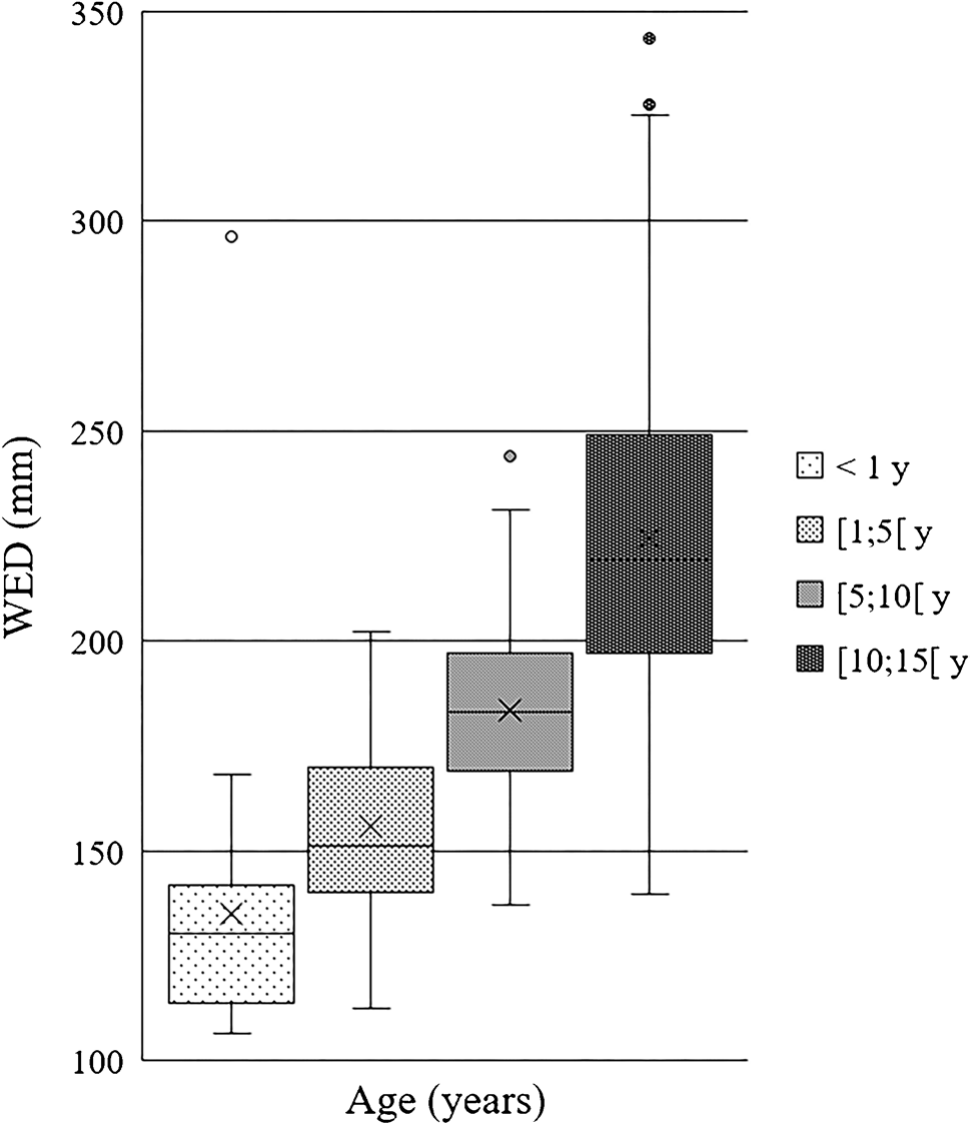
Distribution of water-equivalent diameter (WED) according to patient age groups (in years [y]) shown as a box plot

Chest CT examinations were divided into six different patient size groups with 4-cm water-equivalent diameter bins between 13 and 29 cm, according to Kanal et al. [20]. Minimum and maximum water-equivalent diameters were 11 cm and 35 cm, respectively. There was a strong positive, statistically significant correlation between water-equivalent diameter and CTDI_vol_ (r_s_ = 0.773, *P* < 0.001) and water-equivalent diameter and DLP (r_s_ = 0.802, *P* < 0.001). Scatterplots of water-equivalent diameter against CTDI_vol_ and against DLP are shown in Fig. 1 (CTDI_vol_) and Fig. 2 (DLP). Box plots demonstrate the distribution of water-equivalent diameter according to patient age groups (Fig. 3), showing wide ranges, especially in the age group of 10- to 15-year-old children. Most examinations were in patients of size 17 cm to under 21 cm (*n* = 211 [39%]). Sample sizes and distribution of age and gender within patient size groups are given in Table 3.

**Table 3:** Median values (with interquartile ranges in brackets) for age, gender (absolute values), water-equivalent diameter (WED), and radiation dose metrics (volume computed tomography dose index [CTDI_vol]_), dose-length product (DLP), size-specific dose estimates (SSDE) and effective dose (ED) for the different patient size groups (I-VI) undergoing paediatric chest computed tomography (CT)

Group	WED range (cm)	Number of patients^a^	Number of CT exams (percentage)	Exams on male/female patients	Age (years)	WED (mm)	CTDI_vol_ (mGy)	DLP (mGy·cm)	SSDE (mGy)	ED (mSv) (IRCP 103 [27])
all	total	345	538 (100%)	311/227	8 (4–13)	188 (159–216)	1.3 (0.9–2.2)	30 (19–60)	2.4 (1.7–3.6)	1.3 (0.9–1.8)
I	< 13	22	22 (4%)	11/11	0.6 (0.3–1.5)	121 (113–127)	0.3 (0.2–0.4)	5 (3–7)	0.9 (0.5–1.1)	0.5 (0.3–0.6)
II	[13;17[	116	151 (28%)	80/71	3 (2–6)	150 (142–159)	0.8 (0.7–1.2)	19 (14–25)	1.8 (1.5–2.5)	1.0 (0.7–1.4)
III	[17;21[	142	211 (39%)	133/78	8 (6–11)	189 (181–199)	1.2 (1.0–1.7)	29 (22–44)	2.2 (1.8–3.1)	1.2 (0.9–1.5)
IV	[21;25[	72	97 (18%)	52/45	14 (12–15)	227 (218–241)	2.3 (1.8–3.0)	68 (51–88)	3.6 (2.9–4.6)	1.8 (1.3–2.2)
V	[25;29[	31	42 (8%)	28/14	14 (13–15)	260 (254–268)	3.3 (2.7–4.5)	92 (78–135)	4.5 (3.7–6.2)	1.9 (1.6–3.1)
VI	≥ 29	8	15 (3%)	7/8	14 (14–15)	312 (304–326)	7.0 (6.0–8.0)	216 (188–241)	7.8 (6.7–9.3)	4.0 (3.4–4.5)

^a^ individual patients within patient size groups

*ICRP* International Commission of Radiological Protection

**Fig. 4:**
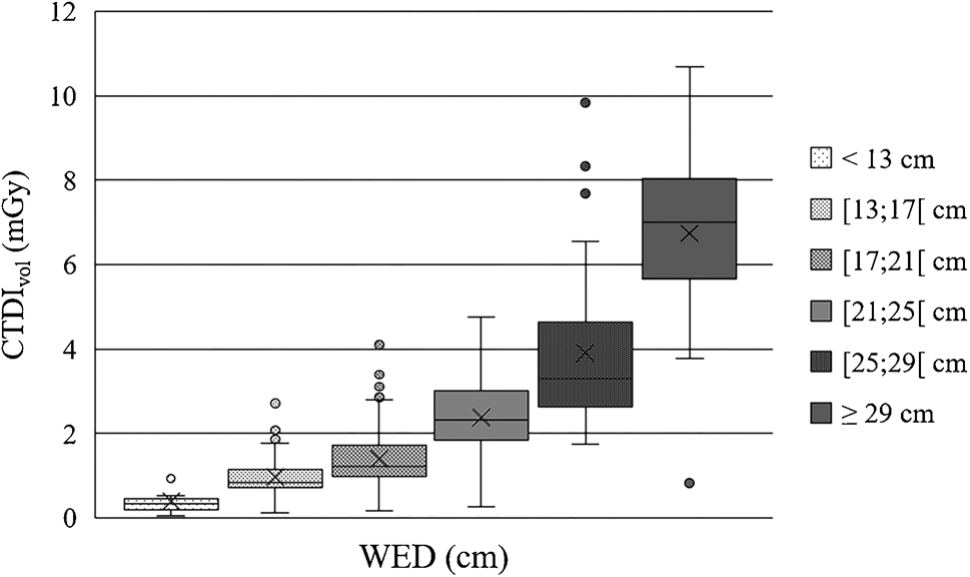
Distribution of volume computed tomography dose index (CTDI_vol_) according to patient size groups determined by water-equivalent diameter (WED) shown as a box plot

**Fig. 5:**
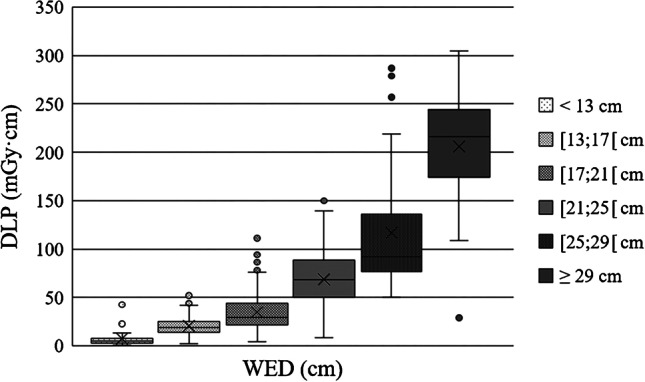
Distribution of dose-length product (DLP) according to patient size groups determined by water-equivalent diameter (WED) shown as a box plot

Distribution of the various dose metrics are also given in Table 3. Dose distributions of CTDI_vol_ and DLP according to the different patient size groups are shown in Fig. 4 (CTDI_vol_) and Fig. 5 (DLP). The Kruskal–Wallis test showed significant differences between patient size groups for all dose metrics (CTDI_vol_, DLP, SSDE, effective dose) (all *P* < 0.001). However, the post hoc test with Bonferroni correction revealed no significant differences between the largest three patient size groups (≥ 21 cm) in terms of CTDI_vol_, DLP and SSDE nor between groups II and III, IV and V and V and VI in terms of effective dose. For all other pairwise comparisons, we mainly observed a moderate to strong effect size (r > 0.3 or r > 0.5).

We calculated achievable doses and DRLs for the six patient size groups (Table 4). Patient size groups were matched to equivalent age and weight groups reported for the national and European DRLs [1, 26]. Local size-based DRLs for CTDI_vol_ were 54% to 74% lower than national DRLs (54% to 71% lower for DLP) and 16% to 75% lower than European DRLs (23% to 85% lower for DLP) for corresponding age and weight groups (Table 5). Local size-based achievable doses for CTDI_vol_ were 72% to 87% lower than national DRLs (75% to 87% lower for DLP) and 50% to 88% lower than European DRLs (56% to 93% lower for DLP).

**Table 4:** Achievable doses (AD) and diagnostic reference levels (DRLs) in terms of volume computed tomography dose index (CTDI_vol_) and dose-length product (DLP) for the different patient size groups (I-VI) in paediatric chest computed tomography

Group	WED range (cm)	CTDI_vol_ (mGy)		DLP (mGy·cm)	
		AD	DRL	AD	DRL
I	< 13	0.3	0.4	5.0	7.3
II	[13;17]	0.8	1.2	19	25
III	[17;21]	1.2	1.7	29	44
IV	[21;25]	2.3	3.0	68	88
V	[25;29]	3.3	4.5	92	135
VI	≥ 29	7.0	8.0	216	241

*WED* water-equivalent diameter

**Table 5:** Comparison of local diagnostic reference levels (DRLs) for paediatric chest computed tomography by patient size group (I-VI) with national and European DRLs show DRLs, age and weight groups and ratios of DRLs for volume computed tomography dose index (CTDI_vol_) and dose-length product (DLP)

Local		Equivalent age group		Equivalent weight group		Local DRL		National DRL		European DRL		Ratio local DRL/ national DRL		Ratio local DRL/ European DRL	
WED range (cm)	Median age (years) (IQR)	National DRL	European DRL	National DRL	European DRL	CTDI_vol_ (mGy)	DLP (mGy·cm)	CTDI_vol_ (mGy)	DLP (mGy·cm)	CTDI_vol_ (mGy)	DLP (mGy·cm)	CTDI_vol_ (mGy)	DLP (mGy·cm)	CTDI_vol_ (mGy)	DLP (mGy·cm)
-	-	< 3 months	< 1 month	3 to < 5 kg	< 5 kg	-	-	1.0	15	1.4	35	-	-	-	-
< 13	0.6 (0.3–1.5)	3 to < 12 months	1 month to < 4 years	5 to < 10 kg	5 to < 15 kg	0.4	7.3	1.7	25	1.8	50	0.26	0.29	0.25	0.15
[13;17]	3.1 (1.9–5.9)	1 to < 5 years	1 month to < 4 years	10 to < 19 kg	5 to < 15 kg	1.2	25	2.6	55	1.8	50	0.44	0.46	0.64	0.51
[17;21]	8 (6–11)	5 to < 10 years	4 to < 10 years	19 to < 32 kg	15 to < 30 kg	1.7	44	4.0	110	2.7	70	0.43	0.40	0.64	0.63
[21;25]	14 (12–15)	10 to < 15 years	10 to < 14 years	32 to < 56 kg	30 to < 50 kg	3.0	88	6.5	200	3.7	115	0.46	0.44	0.81	0.77
[25;29]	14 (13–15)	-	14 to < 18 years	-	50 to < 80 kg	4.5	135	-	-	5.4	200	-	-	0.84	0.67
≥ 29	15 (14–15)	-	-	-	-	8.0	241	-	-	-	-	-	-	-	-

*IQR* interquartile range, *WED* water-equivalent diameter

Technical parameters for chest CT within the different patient size groups are shown in Table 6. The Kruskal–Wallis test showed significant differences between patient size groups for kVp, mean mAs and scan length (all *P* < 0.001). However, the post hoc test with Bonferroni correction revealed no significant differences between the largest three patient size groups (≥ 21 cm) nor between groups I and II in terms of kVp and scan length. The pairwise comparisons for mean mAs mainly showed no significant differences between patient size groups. There was also no significant difference in age between the largest three patient size groups.

**Table 6:** Median values (with interquartile ranges in brackets) for different technical parameters (scan length, tube-voltage peak [kVp], mean tube-current time product [mAs], collimation width, pitch) by patient size group (I-VI) for paediatric chest computed tomography

Group	WED range (cm)	Scan length (mm)	kVp	Mean mAs	Collimation (mm)	Pitch
I	< 13	159 (135–184)	70 (70–80)	52 (31–87)	58 (43–58)	1.9 (1.5–1.9)
II	[13;17[	214 (184–241)	80 (80–100)	69 (29–148)	38 (38–58)	1.9 (0.6–3.0)
III	[17;21[	243 (214–274)	90 (80–100)	40 (25–71)	38 (38–58)	0.6 (0.6–1.7)
IV	[21;25[	293 (259–315)	100 (100–100)	42 (33–56)	38 (38–58)	0.6 (0.6–0.6)
V	[25;29[	287 (270–329)	100 (100–110)	57 (47–69)	38 (38–58)	0.6 (0.6–0.6)
VI	≥ 29	308 (294–320)	100 (100–110)	90 (77–115)	38 (38–58)	0.6 (0.6–0.6)

*WED* water-equivalent diameter

Within patient size groups, the Mann–Whitney *U* test mainly revealed no statistically significant differences for the various dose metrics between examinations performed with and without contrast and only for some groups between low-dose and standard examinations. However, patient size group III showed significant differences for CTDI_vol_ and SSDE between examinations with and without contrast (*P* < 0.05), with lower rank sums for non-contrast examinations. Within this group, non-contrast examinations also showed statistically significant higher values for pitch (*P* < 0.001) because 46% (52 of 112) were performed as high-pitch examinations, and additionally, 63% (70 of 112) were performed using a low-dose technique. Statistically significant differences between low-dose and standard examinations, the latter performed either with or without contrast, were observed for groups III and IV for all dose metrics (all *P* < 0.01) and for group V, except for effective dose (all others in group V, *P* < 0.05), with lower rank sums for low-dose scans. The effect size was small to medium (r < 0.5). No statistically significant difference was observed between examinations with high and standard pitch.

Weight and height were documented in the DICOM data in only 33% and 30% of all examinations, respectively. Within patient size groups, this varied between 14% and 38% for weight. Weight was distributed as follows between patient size groups (median with IQR): I 7.0 kg (6.9 -8.5 kg), II 14.8 kg (12.0–21.0 kg), III 25.0 kg (20.8–33.3 kg), IV 50.0 kg (39.0–56.0 kg), V 61.0 kg (55.0–66.9 kg) and VI 81.0 kg (70.5–95.0 kg). Overall, this agrees with the national and European DRLs for correlating weight groups (Table 5).

## Discussion

Optimising chest CT protocols in children is crucial, particularly since some of the most radiosensitive organs, such as the red bone marrow, breast tissue or thyroid gland are in the direct path of the radiation beam. We established local achievable doses and DRLs for paediatric chest CT as a function of a patient water-equivalent diameter group for children 15 years old and younger. Examinations were performed at a high-volume, multi-site radiology centre on modern multi-slice CT scanners. Our size-based achievable doses and DRLs were much lower than national and European DRLs for corresponding age and weight groups, highlighting the potential for dose reduction in children. This might be explained by the synergetic use of different dose reduction techniques and the use of modern, multi-slice CT scanners, whereas European and national DRLs may reflect average CT practice.

Radiation doses increased with increasing patient size, but no statistical significance was found for the three largest patient sizes. The three largest patient size groups showed no significant differences in age. There were mainly no statistically significant differences within patient size groups between examinations with and without contrast medium, or high-pitch and standard-pitch examinations, and only for the groups III to V between low-dose and standard examinations. The differences in radiation doses may be mainly due to differences in patient size in terms of water-equivalent diameter and technical settings such as applied tube voltage and scan length. The X-ray exposure is approximately proportional to the square of tube voltage. The combination of low tube voltage, which was applied in 180 protocols (tube voltages of 70 kVp and 80 kVp) as well as modern (model-based) iterative reconstruction techniques and automatic dose modulation, both of which were applied to all protocols, may contribute to a significant dose reduction. Furthermore, the high-pitch protocols enable reduction in scan time and free-breathing techniques and may help to reduce motion artefact [28]. This is important especially in younger children and may make sedation unnecessary [28]. Tabari et al. [28] reported no significant difference in SSDE between high-pitch and standard-pitch studies. We also found no significant difference in radiation exposure between high-pitch and standard-pitch protocols within patient size groups (note our low sample sizes within some patient size groups).

Low-dose CT protocols are reported to be well-suited for imaging of high contrast structures, such as lung parenchyma in chest imaging. This may be attributable to a lower X-ray absorption of high contrast structures, which allows a sufficient diagnostic image quality despite high image noise. However, low tube voltage examinations in children are critically discussed as a potential source of increased surface dose due to a higher radiation absorption for superficial, radiosensitive tissues such as the breast [29]. These concerns may be unfounded, as demonstrated by phantom studies showing that the increase in surface dose by low-dose examinations is not significant [30, 31]. The effect of an increased surface dose is reported to be negligible in children, as CTDI_vol_ and noise level are consistent throughout kilovolt peak values [30, 31].

DRLs for adults are often based on standard-size phantoms, standard-size patient groups, or averaged sizes across all patients, whereas for children, DRLs are often based on body weight or age. The European guidelines on DRLs for paediatric imaging recommend specific grouping of patients based on weight (Table 7.1. in [1]) and appropriate age groups to compare weight-based with age-based DRLs (Table 7.2. in [1]). Célier et al. [19] reported DRLs for body examinations as a function of patient age, as weight was available for only 40% of the patients in their study. In our study, weight was available in only 33% of all examinations. Kanal et al. [20] established size-based DRLs and achievable doses based on water-equivalent diameter for adults and for chest CT. We have followed the water-equivalent diameter bins used here and applied them to children. Like Kanal et al. [20], we suggest that water-equivalent diameter is equally or perhaps better able to determine different dose levels in children than age and weight. Especially for chest CT, chest diameter is probably more relevant than weight for radiation dose. However, analysis of weight also showed agreement with patient size groups. To improve future analyses and provide better comparability with other DRLs, we are going to change our institutional requirement so that patient characteristics such as weight and height must be recorded for each examination in all children. We also calculated achievable doses and DRLs for SSDE, which indicates a more realistic patient dose than CTDI_vol_ because SSDE also takes patient size into account.

Since we mainly use modern CT scanners and routinely monitor patient radiation exposure at our institution, we expected low radiation doses. However, the achievable doses and DRLs were far below published national and European DRLs for corresponding age and weight groups. Our data analysis showed that a significant saving in applied radiation exposure is possible. This is of great clinical importance, as children are more sensitive to radiation than adults and the attributable risk of developing cancer due to radiation exposure must be kept as low as possible.

An advantage of our study is that we determined achievable doses and DRLs for paediatric chest CT on different modern CT scanners; a disadvantage is that all scanners are from only one manufacturer. Protocols and radiation doses may differ on CT scanners from other manufacturers and may not be directly comparable to our protocols. For patient size groups II to IV, we had relatively large sample sizes, but patient numbers were small for the marginalized groups. Because of the limited sample size, it was not possible to determine DRLs for different chest protocols, such as chest CT with and without contrast, or indication-based DRLs for different patient size groups, even when CT indications were reported. In addition, we only included patients for whom complete DICOM information was sent to the dose monitoring software. Furthermore, we did not analyse image quality because all examinations were routinely performed at our institution for clinical indications and no common agreement upon image quality criteria exists. Image quality was sufficient to answer the clinical question, and unnecessary repetition of examinations must be avoided. Diagnostic accuracy is always important in all dose optimisation procedures. Radiation exposure should be as low as reasonably achievable, but always sufficient for diagnosis.

## Conclusion

We developed local diagnostic reference levels and achievable doses for paediatric chest CT as a function of patient size. Our size-based diagnostic reference levels and achievable doses can assist other centres with further dose optimisation in children and provide assistance to national authorities responsible for radiation protection who may possibly update paediatric diagnostic reference levels so that they are a function of patient size rather than age or weight. In the future, detailed knowledge of patient characteristics as an input parameter may allow deep learning or intelligent software algorithms to anticipate radiation exposure before the actual CT examination.
